# Cryptogenic stroke and patent foramen ovale (abridged and translated version)

**DOI:** 10.1186/s42466-019-0008-2

**Published:** 2019-02-28

**Authors:** Hans-Christoph Diener, Armin Grau, Stephan Baldus

**Affiliations:** 1 German Society of Neurology, Stuttgart, Germany; 2 DSG German Stroke Society, Berlin, Germany; 3 German Society of Cardiology, Düsseldorf, Germany

**Keywords:** Stroke, Ischemic stroke, Cryptogenic stroke, Echocardiography, Patent foramen ovale (PFO), Closure of patent foramen ovale, Antithrombotic therapy, Anticoagulation, Antiplatelet therapy

## Abstract

Interventional patent foramen ovale (PFO) closure should be performed in patients aged 16 to 60 years (after extensive neurological and cardiological diagnostic work-up) with a history of cryptogenic ischaemic stroke and patent foramen ovale, with moderate or extensive right-to-left shunt.

In patients with cryptogenic ischaemic stroke and patent foramen ovale, who reject a PFO closure, there is no evidence of superiority of oral anticoagulation over antiplatelet therapy. Therefore, secondary prevention should be performed with aspirin or clopidogrel.

Atrial fibrillation, pericardial tamponade, and pulmonary embolism are reported complications during and after implantation of an occluder. However, these events are so rare that they should not influence the recommendation for implantation.

This article is an abridged and translated version of the guideline published in Nervenarzt: Diener, HC., für die Deutsche Gesellschaft für Neurologie (DGN), Grau, A.J. et al. Nervenarzt (2018) 89: 1143. 10.1007/s00115-018-0609-y.

**Electronic supplementary material:**

The online version of this article (10.1186/s42466-019-0008-2) contains supplementary material, which is available to authorized users.

## Need for a recommendation

This article is an abridged and translated version of the guideline published in Nervenarzt: Diener, HC., für die Deutsche Gesellschaft für Neurologie (DGN), Grau, A.J. et al. Nervenarzt (2018) 89: 1143. 10.1007/s00115-018-0609-y.

In stroke patients under 60 years of age with a cryptogenic stroke, for which after extensive diagnostic work up no other potential cause other than a open foramen ovale is detected there are several options for secondary prevention of future strokes. These options include, antiplatelet therapy, anticoagulation and invasive occlusion of the patent foramen ovale (PFO). Previous guidelines gave only low level recommendations for any of these options. Recently, 4 randomized clinical trials on PFO-closure have been published, which changed the evidence base for treatment decisions.

The previous DGN guidelines for the secondary prevention of cryptogenic stroke were published in 2012 and did not reflect the more recent studies on the interventional closure of patent foramen ovale (PFO).

In the meantime, the term cryptogenic stroke has been widely replaced by the criteria of embolic stroke of undetermined source (ESUS) which are applied to patients with cryptogenic stroke [1]. These include:

1. Evidence of cerebral ischaemia by CT or MRI and exclusion of lacunar infarcts
2. Exclusion of large vessel disease of extra- and intracranial arteries (> 50% lumen constriction)
3. Exclusion of other cardiac sources for embolism (e.g. atrial fibrillation) and
4. Exclusion of other stroke mechanisms (e.g. vasculitis, dissection, substance abuse).

## Key questions and main recommendations at a glance

**Question and Recommendation 1**

Does an interventional closure with an occluder reduce the risk for a recurrent stroke, compared with antithrombotic drug therapy, in patients with cryptogenic stroke and patent foramen ovale (PFO) with or without atrial septal aneurysm?

**Interventional PFO closure should be performed in patients aged 16 to 60 years (after extensive neurological and cardiological diagnostic work-up) with a history of cryptogenic ischaemic stroke and patent foramen ovale, with moderate or extensive right-to-left shunt.**

*Recommendation level A, Evidence level I.*

**Question and Recommendation 2**

Does the administration of antiplatelet therapy reduce the risk of recurrent stroke in patients with cryptogenic stroke and PFO with or without atrial septal aneurysm when compared with oral anticoagulation?

**In patients with cryptogenic ischaemic stroke and patent foramen ovale, who reject a PFO closure, there is no evidence of superiority of oral anticoagulation over antiplatelet therapy. Therefore, secondary prevention should be performed with aspirin or clopidogrel.**

*Recommendation level B, Evidence level II.*

**Question and Recommendation 3**

Which antithrombotic therapy should be used for the period after an interventional PFO closure?

**In patients with cryptogenic ischaemic stroke and patent foramen ovale, who reject a PFO closure, there is no evidence of superiority of oral anticoagulation over antiplatelet therapy. Therefore, secondary prevention should be performed with aspirin or clopidogrel.**

*Recommendation level B, Evidence level IIb.*

**Question and Recommendation 4**

Is the interventional closure of a patent foramen ovale associated with side effects (other than bleeding complications) in patients with cryptogenic stroke compared to antithrombotic therapy?

**Atrial fibrillation, pericardial tamponade, and pulmonary embolism are reported complications during and after implantation of an occluder. However, these events are so rare that they should not influence the recommendation for implantation.**

*Recommendation level A, Evidence level Ia.*

**Question and Recommendation 5**

Are there differences in the rate of closure and complication rates between the used closure systems?

**Disc occluders were found to be superior to non-circular disc occluders in terms of safety and effectiveness.**

*Recommendation level A, Evidence level Ia.*

## Epidemiology

Autopsy studies and echocardiographic studies show that a patent foramen ovale (PFO) is present in between 20 and 25% of the healthy population. In younger stroke patients, the prevalence is up to 45%. In particular, patients younger than 55 years with a PFO are at increased risk of cryptogenic ischaemic stroke [2]. A meta-analysis of 14 prospective studies with 4251 patients with stroke who were conservatively treated showed that patients with and without PFO were at almost equal risk of recurrent stroke or TIA (RR = 1.18, 95% CI 0.78–1.79) [3].

## Recent randomised studies

There are several older randomised studies, which compared an interventional closure of patent foramen ovale in patients with cryptogenic stroke, with drug therapy only [4–6] (Table 1).

**Table 1:** Design and baseline data from the six randomised studies on PFO closure in cryptogenic stroke [9, 11, 23]

Parameters	Closure I	PC Trial	RESPECT^a^	REDUCE	CLOSE	Defense PFO
Patients (n)	909	414	980	664	663	120
Mean age	46	44.5	46	42.2	43.3	51.8
RLS (%)	53	65.6	48.8	81.3	100	53
ASA (%)	36.6	23.7	35.7	20.6	32.8	10
ATH	ASA, OAC	APT, OAC	APT, OAC	APT	APT, OAC	APT
OAC (%)	34	31	25	0	28	0
Device	STARFlex	Amplatzer PFO	Amplatzer PFO	Cardioform Helex	no specification	Amplatzer
Endpoint	Stroke, TIA, death	Death, stroke, TIA, embolism	Stroke, early death	Stroke	Stroke	Stroke, vascular death, TIMI bleeding
Follow-up (months)	44	49	70.8	38.4	63.6	24

*RLS* moderate or major right-to-left shunt, *ASA* atrial septal aneurysm, *ATH* antithrombotic therapy, *ASA* acetylsalicylic acid, *APT* antiplatelet therapy, *OAC* oral anticoagulation

^a^Data refer to the second evaluation of the study at 5.9 years [9]

The study “Closure or Medical Therapy for Cryptogenic Stroke with Patent Foramen Ovale” (CLOSURE-I) randomised 909 patients with cryptogenic stroke or transient ischaemic attacks (TIA) within the last 6 months and aged between 18 and 60 years into one therapy arm with interventional closure of patent foramen ovale (using STARFlex® occluder formerly NMT Medical) or drug treatment only [4]. The primary endpoint was the frequency of strokes or TIA in the two-year follow-up period, all-cause mortality in the first 30 days, or death due to neurological causes between day 31 and 2 years. The primary endpoint was achieved by 5.5% of the patients in the intervention group and 6.8% in the conservative treatment group. This difference with a relative risk reduction of 22% was not statistically significant with a hazard ratio (HR) of 0.78, (95% confidence interval 0.45–1.35) and a *p*-value of 0.37. However, the initial planned sample size for this study was reduced several times for financial reasons.

The study “Percutaneous Closure of Patent Foramen Ovale in Cryptogenic Embolism” (PC trial) studied 414 patients aged 18 to 60 years with cryptogenic ischaemic stroke, TIA (with tissue lesion in imaging = stroke as per new TIA definition) or peripheral embolism [5]. Patients had either an interventional closure of the patent foramen ovale with the Amplatzer occluder (Abbott) or drug treatment only with antiplatelet therapy or oral anticoagulation. The observation period was 4 years. The primary endpoint was a composite endpoint of death, non-fatal stroke, TIA, or peripheral embolism. The primary endpoint occurred in 3.4% of patients in the PFO closure group and in 5.2% in the conservative treatment group. The difference was statistically not significant with a HR of 0.63, (95% confidence interval 0.24–1.62, *p* = 0.34).

The study “Closure of Patent Foramen Ovale versus Medical Therapy after Cryptogenic Stroke” (RESPECT) randomised 980 patients aged 18 to 60 years with cryptogenic ischaemic stroke in the previous 9 months, into an interventional closure group (Amplatzer occluder) of the patent foramen ovale compared to drug treatment with antiplatelet therapy or anticoagulants [7]. The primary endpoint was the combination of stroke during the follow-up period (planned: 2 years) or early all-cause death (intervention group: within 30 days post surgery or 45 days after randomisation, whichever occurs later; conservative group: 45 days after randomisation. In the intention-to-treat (ITT) analysis, 9 events occurred in the group of patients who received interventional PFO closure, compared to 16 events in the conservative treatment group. This difference was not significant with an HR of 0.49 and a 95% confidence interval of 0.22–1.11 and a *p*-value of 0.08.

In the per-protocol analysis the difference was significant with a HR of 0.37 and a p-value of 0.03. Three cerebral events occurred in the group of subjects randomised to the interventional closure group before the implantation of the occluder.

In summary, the older studies in the ITT analysis over a relatively short follow-up period do not show superiority of PFO closure versus drug therapy for their primary endpoints. However, in each intervention group, the event rate was numerically lower than in the drug treatment arm. In the RESPECT study, a significant event reduction was observed in the occluder group in the per-protocol analysis.

Three more recent studies published in 2017 and one study in 2018 study demonstrated the efficacy of PFO closure in patients with cryptogenic stroke < 60 years for the reduction of recurrent stroke.

The three-arm CLOSE study examined whether in patients 16 to 60 years of age with a patent foramen ovale and cryptogenic stroke, PFO closure is superior to anticoagulation or treatment with antiplatelet therapy (APT) [7]. This was a multicentre, randomised, open-label study in which patients were randomised at a 1:1:1 ratio to PFO closure with APT, treatment with APT as monotherapy, or oral anticoagulation. The stroke without other competing aetiologies had to have occurred within the last 6 months. Inclusion criterion was a patent foramen ovale with an atrial septal aneurysm or a large interatrial shunt. The mean patient age was 43.3 years and they were mostly men. Two-thirds of patients had a large shunt volume without atrial septal aneurysm. Patients who had a contraindication to oral anticoagulation were randomised to either PFO closure or APT treatment. Patients with a contraindication to interventional PFO closure were randomised to either anticoagulation or APT therapy. In patients with PFO closure, dual antiplatelet therapy was performed for 3 months with acetylsalicylic acid and clopidogrel followed by monotherapy. The primary endpoint was fatal and non-fatal stroke. Secondary endpoints were the combination of transient ischaemic attacks (TIA) and systemic embolism. A total of 663 patients were enrolled in the study. 238 patients received PFO closure, in the majority with an Amplatzer occluder, 335 patients were treated with APT, and 187 patients were anticoagulated. The mean observation time was 5.3 years. In this observation time, the group with PFO closure had no stroke in 238 patients, compared with 14 strokes in 235 patients in the group treated with APT. This corresponds to an HR of 0.03 with a 95% confidence interval of 0–0.26 and a *p*-value of less than 0.001. Complications occurred in 14 patients (5.9%) of the PFO closure group, including new-onset atrial fibrillation. When patients without PFO closure were compared, there were 3 strokes in 187 patients in the anticoagulation group and 7 strokes in 147 patients in the APT group (HR anticoagulation vs APT = 0.44 (95% confidence interval 0.11–1.48), *p* = 0.18). A (transient) atrial fibrillation/flutter was significantly more common in the closure group (4.6% vs 0.9%, *p* = 0.02). The study was funded by the French Ministry of Health. In summary, the CLOSE study shows a therapeutic benefit of PFO closure versus drug therapy alone in a patent foramen ovale with large shunt volume or atrial septal aneurysm in patients aged below 60 years with cryptogenic stroke, and no difference between anticoagulation and APT.

The REDUCE study compared the closure of a patent foramen ovale with subsequent APT, with therapy with APT alone in patients with patent foramen ovale and cryptogenic stroke [8]. The PFO closure was performed with the Gore HELEX or Cardioform Septal occluder. This was a multinational study, in which patients aged 18 to 59 years with cryptogenic stroke, as well as an echocardiographic proven right-to-left shunt or an atrial septal aneurysm were enrolled. The mean patient age was 42.2 years. In approximately 20% of the patients the shunt volume was small; in 40%, it was moderately sized; and in 40% the shunt volume was large. 20% had an atrial septal aneurysm. Co-primary endpoints were 1) the absence of clinical evidence of recurrent ischaemia and 2) the incidence of new strokes (incidence of clinically apparent or silent cerebral ischaemia in cerebral imaging). Imaging was performed at enrolment and after 2 years by nuclear magnetic resonance imaging.

Six hundred sixty four patients were enrolled. During the median follow-up of 3.2 years, ischaemic stroke recurred in 6 of 441 (1.4%) patients in the PFO closure group and 12 of 223 (5.4%) patients in the APT group. This corresponds to an HR of 0.23 (95% confidence interval of 0.09–0.62) which was significant with a *p*-value of 0.002. In combination with cerebral imaging, 22 patients (5.7%) in the PFO closure group and 20 patients (11.3%) in the control group had a new cerebral ischaemia (defined as clinical stroke or silent lesion). This corresponds to a relative risk of 0.51 (95% confidence interval of 0.29–0.91) and a p-value of 0.04. However, clinically silent infarctions alone were not significantly different between the two treatment groups. Six (1.4%) patients experienced serious complications due to the occlusion system and 29 (3.6%) patients in the PFO closure group experienced at least transient atrial fibrillation.

The REDUCE study shows a significant benefit of PFO closure in patients aged below 60 years with cryptogenic stroke and large right-to-left shunt or atrial septal aneurysm. New silent infarctions occurred in the cerebral MRI imaging with similar frequency in both groups.

The long-term results of the RESPECT study (see above) were also published in 2017 [9]. The multicentre randomised open-label study included patients aged 18 to 60 years with cryptogenic stroke and a patent foramen ovale. Patients received either PFO closure with the Amplatzer occluder, followed by acetylsalicylic acid plus clopidogrel for 1 month, and monotherapy with acetylsalicylic acid for 5 months. In the conservative treatment group, patients were treated with acetylsalicylic acid, clopidogrel, acetylsalicylic acid plus extended-release dipyridamole or warfarin. The primary endpoint was a composite endpoint of new fatal or non-fatal ischaemic stroke or early death within 30 days following implantation of the closure system or 45 days after randomisation.

Nine hundred eighty patients with a mean age of 46 years were enrolled. The median observation time was 6 years. Ischaemic stroke recurred in 18 of 499 (3.6%) patients in the PFO closure group and 28 of 481 (5.8%) patients in the drug-treatment group. This corresponds to an HR of 0.55 (95% confidence interval of 0.31–0.999) which was significant with a *p*-value of 0.046. Ischaemic stroke of undetermined origin recurred in 10 patients in the PFO closure group and 23 patients in the drug-treatment group. This corresponds to an HR of 0.38 (95% confidence interval of 0.18–0.79) and a p-value of 0.007. Subgroup analyses showed that the treatment difference is independent of age and gender. Patients with a large shunt volume and atrial septal aneurysm had greater therapeutic efficacy of the PFO closure. This was also the case for patients treated with APT instead of anticoagulation.

Like the other more recent randomised studies, the long-term results of the RESPECT study show that the interventional closure of a patent foramen ovale is superior to antithrombotic therapy in patients with cryptogenic stroke aged below 60 years and a large right-to-left shunt or atrial septal aneurysm.

The Korean DEFENSE PFO study randomly assigned 60 patients with PFO and cryptogenic stroke each to a PFO closure or drug treatment [10]. Inclusion criteria were a PFO with atrial septal aneurysm or a PFO with a size > 2 mm. The primary endpoint was the composite of stroke, vascular death, and major bleeding according to TIMI criteria. The primary endpoint occurred in 6/60 patients during the 2-year observation period in the group of purely drug-treated patients. This corresponds to a 2-year event rate of 12.9%. The primary endpoint did not occur in the PFO closure group (*p* = 0.013). Complications of the PFO closure included atrial fibrillation (2 x), pericardial effusion (1 x) and a pseudoaneurysm at the puncture site (1 x).

## Key question 1

**Does an interventional closure with an occluder reduce the risk for a recurrent stroke, compared with antithrombotic drug therapy, in patients with cryptogenic stroke and patent foramen ovale (PFO) with or without atrial septal aneurysm?**

If the results of the six randomised studies are combined (Table 2) a relative 75% reduction for the stroke endpoint in favour of PFO closure is found. However, this applies only to patients aged < 60 years and with a medium to large right-to-left shunt. Whether patients older than 60 years or with a smaller shunt benefit from an occluder implant has not yet been adequately studied. The “number needed to treat” (NNT) to prevent an event in 3.7 years is 47 [11].

**Table 2:** Results from the six randomised studies on PFO closure in cryptogenic stroke (data from 10, 12, 21)

Parameters		Closure I	PC Trial	RESPECT	REDUCE	CLOSE	Defense PFO
Stroke (%)	M	3.1	2.4	5.8	5.4	6.0	10.5
Stroke (%)	C	2.9	0.5	3.6	1.4	0.0	0
TIA (%)	M	4.1	3.3	4.8	–	–	2.0
TIA (%)	C	3.1	2.5	3.4	–	–	0
Death (%)	M	0	0	2.2	0	0	0
Death (%)	C	0	1.0	1.4	0.5	0	0

*M* medical treatment; *C* PFO Closure

Until the end of April 2018, the results of 6 meta-analyses were available on the closure of patent foramen ovale in patients with cryptogenic stroke [11–15]. Results are shown in Table 3.

**Table 3:** Results of meta-analyses for PFO closure in cryptogenic stroke

Author	Studies	N	Endpoint		95% CI	P
Abo Salem et al. [12]	5	3627	Stroke	RR 0.48	0.3–0.7	0.001
De Rosa et al. [13]	4	3216	Stroke and TIA	RD −0.029	−0.050 - -0.007	0.008
Ntaios et al. [11]	5	3627	Stroke	OR 0.43	0.21–0.90	NI
Shah et al. [14]	4	2892	Stroke	RD −0.032	−0.050 - -0.014	0.011
Saver et al. [24]	6	3560	Stroke	HR 0.30	0.13–0.68	0.004
Ahmad et al. [15]	5	3440	Stroke	HR 0.32	0.13–0.82	0.018

*RR* relative risk; *CI* confidence interval, *RD* Risk Difference OR Odds Ratio, *NI* not indicated

However, it must be considered that one of the limitations is that the number of disabling strokes in the studies was very low or not published. Furthermore, patients who meet the criteria for cryptogenic stroke or ESUS frequently have additional vascular risk factors that may potentially contribute to stroke via different pathomechanisms than a PFO. The ROPE score [16] is a helpful tool to detect the likely role of PFO in patients with unexplained stroke aetiology and PFO. A high ROPE score (0–10 points) supports a causal significance of the PFO. The ROPE score (> ≠ 7 vs < ≠ 7) was studied in the CLOSE study in a pre-specified subgroup analysis. Patients had a mean ROPE score of 7, indicating a good selection of patients. Results from subgroup analysis are not yet available.

Also, it is important to consider that the risks of the PFO closure may be higher under day-to-day conditions than under study conditions and that the long-term PFO closure risks are not known.

**Recommendation 1**

**Interventional PFO closure should be performed in patients aged 16 to 60 years (after extensive neurological and cardiological diagnostic work-up) with a history of cryptogenic ischaemic stroke and patent foramen ovale, with moderate or extensive right-to-left shunt.**

***Recommendation level A, Evidence level I.***

## Key question 2

**Does the administration of antiplatelet therapy reduce the risk of recurrent stroke in patients with cryptogenic stroke and PFO with or without atrial septal aneurysm when compared with oral anticoagulation?**

In the CLOSURE-I study, 441 patients were treated with warfarin (INR 2.0–3.0) (*n* = 111), acetylsalicylic acid 325 mg (*n* = 252), or both (*n* = 40) in the drug therapy group [4]. 38 patients did not receive antithrombotic therapy. No statistically significant differences between the four treatment groups (7.9, 6.7, 5.4, 5.9%) were observed for the primary endpoint (incidence of strokes or transient ischaemic attacks in the two-year follow-up period, all-cause mortality in the first 30 days, or death by neurological causes between day 31 and 2 years).

In the PC study, the antithrombotic therapy in the drug-treatment group was left to the decision of the attending physicians. Oral anticoagulation or APT therapy was recommended. After 6 months, 10.5% of patients were treated with oral anticoagulation, 58% with aspirin, 10.5% with clopidogrel, and 5% remained untreated. There was no difference in the rate of events under anticoagulation and APT (HR = 0.2; 95% confidence interval 0.03–1.61) [17].

In the RESPECT study, patients were able to receive treatment with aspirin, clopidogrel, warfarin, or aspirin plus extended release dipyridamole [9]. The incidence of recurrent stroke in the 481 patients in the conservative treatment group was not different between the 360 patients who received APT (6.4%) and the 121 patients who received anti-coagulant (4.1%).

In the REDUCE study, aspirin (75 or 325 mg), aspirin plus dipyridamole, or clopidogrel (75 mg) could be used in the conservative therapy group [8]. Since oral anticoagulation was not allowed, a comparison between anticoagulation and APT cannot be made.

In the CLOSE study, patients were randomised to a group with oral anticoagulation (vitamin K antagonist or NOAC) or APT (aspirin, aspirin plus dipyridamole, clopidogrel) [7]. There was no difference between the anticoagulation group (3/187) and APT (7/174) for the “recurrent stroke” endpoint. The frequency of serious bleeding complications was not significantly higher with anticoagulation (10/187) than with APT (4/174).

In a propensity score matching analysis comparing secondary prevention with oral anticoagulation and APT, 2385 patients were evaluated from multiple observational studies and the CLOSURE, RESPECT and PC trial, 804 with anticoagulation, and 1581 with APT [17]. Both treatments were equally effective in this analysis.

The numerically slightly increased rate of bleeding with anticoagulation, the anticipated long duration of secondary prophylaxis, and an overall low probability of recurrent stroke support APT as the preferred treatment choice. No statement can be made for direct non-vitamin K antagonists (DOACs) because appropriate data are missing.

**Recommendation 2**

**In patients with cryptogenic ischaemic stroke and patent foramen ovale, who reject a PFO closure, there is no evidence of superiority of oral anticoagulation over antiplatelet therapy. Therefore, secondary prevention should be performed with aspirin or clopidogrel.**

***Recommendation level B, Evidence level II.***

## Key question 3

**Which antithrombotic therapy should be used for the period after an interventional PFO closure?**

In the CLOSURE-I study, patients received 75 mg clopidogrel for 6 months after the PFO closure and aspirin 81 or 325 mg for 2 years [4]. Serious bleeding was not significantly more common under this therapy (10/378 = 2.6%) than in the drug-only therapy group (4/374 = 1.1%) (*p* = 0.11). In the PC study, patients received 100 or 325 mg of aspirin for 5–6 months plus ticlopidine (250 or 500 mg) or clopidogrel (75 or 150 mg) for 1–6 months after PFO closure [5]. Serious bleeding was not different between the PFO closure group (2/204 = 1.0%) and the conservative therapy group (2/210 = 1.0%). In the RESPECT study, patients following PFO closure were treated with 75 mg clopidogrel for 1 month, and for 6 months with aspirin (81 or 325 mg) [9]. The frequency of bleeding complications did not differ between the PFO closure group (3/499 = 0.6%) and the conservative therapy group (1/481 = 0.2%) (*p* = 0.624). In the REDUCE study, patients received aspirin (75 or 325 mg), aspirin plus dipyridamole, or clopidogrel (75 mg) [8].

Patients in the PFO closure group received an additional 300 mg of clopidogrel on the day of intervention and then 75 mg for 3 days. Serious bleeding occurred in 8/441 (1.8%) in the intervention group and 6/223 (2.7%) in the conservative treatment group (*p* = 0.57). In the CLOSE study, after PFO closure, patients received a dual platelet therapy with 75 mg of clopidogrel and 75 mg of aspirin for 3 months followed by monotherapy for the remainder of the study. Serious or fatal bleeding occurred in 2/238 (0.8%) patients in the PFO closure group and 5/235 (2.1%) patients treated with APT (*p* = 0.28) [7].

In summary, most studies performed dual platelet inhibition over a period of 1–6 months after PFO closure, followed by monotherapy for 6 months up to 2 years. Statistical comparison of the different regimens is not possible. We recommend antiplatelet therapy of 12–24 months in patients in whom the PFO is the only potential source of embolism. In patients with cryptogenic stroke and PFO and other competing stroke mechanisms (e.g. atherosclerosis), we recommend long-term prophylaxis with platelet inhibitors.

**Recommendation 3**

**After an interventional PFO closure, dual antiplatelet therpapy is recommended with 100 mg aspirin plus 75 mg clopidogrel for 1–3 months, followed by 12–24 months of monotherapy with 100 mg aspirin or 75 mg clopidogrel. In patients with additional manifestation of atherosclerosis, long-term therapy with antiplatelet therapy is recommended.**

***Recommendation level B, Evidence level IIb.***

## Key question 4

**Is the interventional closure of a patent foramen ovale associated with side effects (other than bleeding complications) in patients with cryptogenic stroke compared to antithrombotic therapy bitte fett?**

The overall rate of serious adverse events was not significantly increased in any of the randomised post-interventional PFO closure studies (Table 4) [4, 5, 7–9].

**Table 4:** Adverse events in the six randomised studies of PFO closure in cryptogenic stroke

Parameters	Group	Closure I	PC Trial	RESPECT	REDUCE	CLOSE	Defense PFO
Device		STARFlex	Amplatzer PFO	Amplatzer PFO	HELEX/Cardioform	Different devices	Amplatzer PFO
1. General Adverse Events							
	M	16.9	17.6	40.3	27.8	33.2	–
Overall SAE (%)	C	16.6	21.1	36.0	23.1	35.7	–
	P	0.90	0.37	0.17	0.22	0.56	–
Atrial fibrillation or flutter (%)	M	0.7*	1.0	1.5	0.4*	0.9*	0
	C	5.7*	2.9	3.0	6.6*	4.6*	3.3
	P	< 0.001	0.17	0.13	< 0.0001	0.02	–
Overall major bleeding (%)	M	1.1	1.4	0.2	2.7	2.1	4.9
	C	2.6	0.5	0.6	1.8	0.8	0
	P	0.11	0.62	0.6	0.57	0.28	0.15
	M	0	0	0.6*	0.4	0	0
Pulmonary embolism (%)	C	0	0	2.4*	0.5	0.4	0
	P	–	–	0.034	1.0	–	–
2. Procedural and occluder-associated complications							
Atrial fibrillation or flutter (%)	C	3.5	0.5	1.4	5.4	4.2	3.0
Bleeding (%)	C	2.5	1	0.6	0.9	0	0
Pericardial tamponade (%)	C	0	0	0.4	0.2	0	0
Cardiac thrombus (%)	C	1.1	0	0.2	0.5	0.4	0
Cardiac perforation (%)	C	0.25	0	0.2	0	0	0
Occluder embolisation (%)	C	0	0	0	0.7	0	0
Rare complications classified as procedural or occluder-associated (%)	C	0.5^a^	0	2.8^b^	2.3^c^	1.7^d^	3.3^e^

SAE = serious adverse events; M = medical treatment; C = PFO Closure

*significant difference for *p* < 0.05

^a^1 x peripheral nerve lesion and 1 x vessel injury requiring surgical intervention

^b^1 x pericardial effusion without tamponade, 1 x allergic drug reaction, 1 x vasovagal reaction, 2 x ischaemic stroke, 1 x chest tightness, 1 x infectious endocarditis, 2 x pulmonary embolism, 1 x deep vein thrombosis, 2 x residual shunt requiring re-closure, 1 x sepsis, 1 x transient ventricular tachycardia

^c^1 x aortic dissection, 1 x AV fistula, 2 x hypotension, 1 x anxiety, 1 x chest tightness, 1 x non-cardiac chest pain, 1 x fatigue, 1 x hemiparesis, 1 x respiratory arrest

^d^2 x supraventricular tachycardia, 1 x air embolism, 1 x hyperthermia

^e^1 x pericardial effusion without tamponade, 1 x ischaemic stroke

Noteworthy side effects were new-onset atrial fibrillation, procedural bleeding events near the catheter insertion site in the groin, dislocation of the occluder, thrombus formation, cardiac perforation, and pericardial effusion/tamponade, with no effect on all-cause mortality compared with the drug-treated group in any of the studies.

The risk of new-onset atrial fibrillation depends on the type of occluder; the relative risk is quantified at 1.33–7.67, with a follow-up of the studies between 2.1 and 5.9 years [18]. Overall, the occurrence of this arrhythmia is low though. The overall incidence following PFO closure was 4.2% in the five large randomised trials [12]. If only disc occluder systems are considered, the risk is not statistically significantly increased compared to drug therapy [17]. Atrial fibrillation was mainly periprocedural in 61% [4] and 91% [7] within 30 days of intervention. The duration of the episodes is relatively short; in 72% of cases, the atrial fibrillation ended spontaneously within 45 days [11]. Patients with pre-procedural evidence of atrial fibrillation were allowed to be enrolled in the studies, but the stringency of the evaluation in this respect was not defined in any study. In addition, it should be noted that no pre- and post-procedural systematic monitoring of the heart rhythm has not been performed in any of the studies (e.g. by regular long-term ECG tests).

The long-term follow-up of the RESPECT study found a significantly higher rate of pulmonary embolism after PFO closure ((12/499 (2.4%) vs 3/481 (0.6%), *p* = 0.034)). This might be due to the fact that the antithrombotic therapy in the intervention group was less intense (oral anticoagulation in 2% after intervention vs 19% in the control group) [9]. In the REDUCE study, pulmonary embolism occurred equally frequently in both groups (0.5% vs 0.4%, *p* = 1.0) and no significant difference in incidence of pulmonary embolism was reported in any of the other major studies [4, 5, 7, 8].

No significant difference was observed between the two groups in the overall rate of major bleeding in either of the studies [4, 5, 7–9].

In the RESPECT study, the incidence of non-procedural bleeding events was not different in the maintenance period (median interval 5.9 years) between patients in the PFO arm and with medication alone (*p* = 0.168). In the REDUCE study, the risk of major bleeding was numerically higher at 2.7% in the APT arm than in the group of patients treated with closure (0.9%, *p* = 0.09) [8]. In the CLOSE study, bleeding rates in patients with OAC and APT were not statistically significantly higher at 2.1 and 5.3%, respectively, compared with the PFO arm (0.8%) [7].

The rate of periprocedural bleeding complications ranged from 0.6 to 2.5% (with minor bleeding, including haematoma at the puncture site) [4, 5, 7–9].

In the randomised studies, cardiac thrombi were observed in 0–1.1% of subjects following PFO closure [4, 5, 7–9]. In the CLOSURE and RESPECT study, cardiac perforation (0.25 and 0.2%) occurred [4, 9], whilst a total of four cases of pericardial tamponades were reported (two in the RESPECT study and one each in the REDUCE study and the DEFENSE PFO study) [8–10]. Three occluder dislocations (0.7%) occurred in the REDUCE study [8]. In the RESPECT study, periprocedural aortic dissection and occluder-associated endocarditis occurred (0.2% each) [9].

**Recommendation 4**

**Atrial fibrillation, pericardial tamponade, and pulmonary embolism are reported complications during and after implantation of an occluder. However, these events are so rare that they should not influence the recommendation for implantation.**

***Recommendation level A, Evidence level Ia.***

## Key question 5

**Are there differences in the rate of closure and complication rates between the used closure systems?**

The six large randomised trials compared mainly the STARFlex occluder, the Amplatzer PFO occluder, and the HELEX occluder with drug-only treatment (some other occluders were used only in the CLOSE study) [4, 5, 7–9]. These three closure systems are the only ones compared directly against each other in a randomised study [19]. Hornung and colleagues randomly assigned 660 patients to the three listed closure systems and compared them for efficacy and safety. In all cases, the implantation was successfully completed. Five years after implantation, the rate of closure ranged from 96.8 to 100%. It was lower in the HELEX occluder (*p* = 0.004) than the other two systems, where the implantation of a second occluder for the closure of a relevant residual shunt was required more frequently (6.8% vs 3.2% with the STARFlex and 0.9% with the Amplatzer occluder, *p* = 0.0038). Furthermore, more embolisms of the system were observed with the HELEX occluder (1.4% vs 0% with the other systems, *p* = 0.049). Atrial fibrillation was significantly more common in the STARFlex occluder compared with the other closure systems (12.3% vs 3.6% for Amplatzer and 2.3% for HELEX occluder, *p* <  0.001). Additionally, more occlusion-associated thrombi (5% vs 0% with Amplatzer and 0.5% with the HELEX system, *p* <  0.001) occurred with the STARFlex occluder. The Amplatzer occluder was superior to the other two devices regarding prevention of the composite primary endpoint (recurrent cerebral ischaemia, death from neurological cause or other paradoxical embolism) (1.4% vs. 5.9% for the STARFLEX device, and 4.1% for the HELEX device, *p* = 0.042).

Stortecky and colleagues compared the three listed occluders in a network meta-analysis, based on the results of the three older randomised trials (CLOSURE, PC trial and RESPECT), and the work of Hornung et al. [18]. Successful implantation was achieved in 89.4 to 100% of the procedures (Amplatzer 95.9–100%, STARFLEX: 89.4–100%, HELEX, 100%). After 6 months, the rate of closure was between 85.9 and 95.9% (Amplatzer: 93.5–95.9%, STARFLEX: 86.1–94.5%, HELEX, 85.9%). The atrial fibrillation risk was significantly higher for the STARFlex system than for the other two devices. Compared with drug therapy, the rate of ischaemic strokes after PFO closure was significantly lower only when using the Amplatzer occluder. The Amplatzer system was most effective in preventing ischaemic strokes compared with the two other closure systems and also compared to the drug therapy alone. Longer follow-up also leads to an increase in recorded closure rates.

In summary, these data suggest that the Amplatzer occluder is superior to the STARFlex and HELEX device both in terms of effectiveness and safety. Ultimately, it must be stated that the data on the comparison of the different occluders is very limited overall, and so far, no randomised data exist for other closure systems available on the market [19].

**Recommendation 5**

**Disc occluders were found to be superior to non-circular disc occluders in terms of safety and effectiveness.**

***Recommendation level A, Evidence level Ia.***

## Additional file

Additional file 1: Statement of competing interests: tabular summary. (DOCX 22 kb)

## Abbreviations

ASA: Acetylsalicylic acid
ASA: Atrial septal aneurysm
APT: antiplatelet therapy
ATH: Antithrombotic therapy
CI: Confidence interval
CT: Computertomography
DGK: German Society of Cardiology
DGN: German Neurological Society
DSG: German Stroke Society
ESUS: Embolic stroke of undetermined source
HR: Hazard ratio
ITT: Intention to treat
M: Medical treatment
MRI: Magnetic resonance imaging
NI: Not indicated
OAC: Oral anticoagulation
PFO: Patent foramen ovale
RD: Risk difference
RLS: Right left shunt
ROPE: Risk of paradoxical embolism
RR: Relaive risk
SAE: Serious adverse event
TIA: Transient ischemic attack

## Editorial guideline committee (alphabetically)

Stephan Baldus, Director, Department of Cardiology, Angiology, Pneumology and Internal Intensive Medicine, Cardiac Centre at University Hospital Cologne, Kerpener Straße 62, 50937 Cologne, Representative of the DGK.

Hans-Christoph Diener, Senior Professor for Clinical Neuroscience, Department of Neurology and Stroke Centre, University Hospital Essen, Hufelandstraße 55, 45147 Essen, h.diener@uk-essen.de, Representative of the DGN.

Alexander Ghanem, Head of Department, Department of Cardiology, Asklepios Hospital St Georg, Lohmuhlenstrasse 5, 20099 Hamburg, Representative of the DGK.

Armin J. Grau, Director of the Neurology Department, Hospital of the City of Ludwigshafen a. Rh., Bremserstrasse 79, 67063 Ludwigshafen a. Rh., gray@klilu.de, Representative of the DSG.

Klaus Gröschel, Department and Outpatient Department of Neurology, University Hospital Mainz, Langenbeckstrasse 1, 55131 Mainz, Klaus.Grosterel@unimedicine-mainz.de, Representative of the DSG.

Christoph Liebetrau, Executive Director of Cardiology, Head of Unit, Cardiac Catheter Laboratory Kerckhoff-Klinik Gmbh Cardiology Department, Benekestraße 2–8, 61231 Bad Nauheim, Representative of DGK.

Steffen Massberg, Medical Department and Outpatient Department I, University Hospital Munich, Ludwig Maximilian University of Munich, Marchioninistrasse 15, 81377 Munich, steffen.massberg@med.uni-muenchen.de, Representative of the DGK.

Helge Möllmann, Senior Consultant of the Department of Internal Medicine, St Johannes Hospital, Johannesstraße 9–13, 44137 Dortmund, Representative of the DGK.

Holger Nef, Medical Department I, Internal Medicine/Cardiology, Klinikstraße 33, 35392 Gießen, Representative of the DGK.

Dirk Sander, Senior Consultant of the Neurology Department, Benedictus Krankenhaus Tutzing Gmbh & Co. KG, Academic Teaching Hospital of the TU Munich, Bahnhofstraße 5, 82327 Tutzing, Dirk.Sander@artemed.de, Representative of the DSG.

Christian Weimar, Department of Neurology, University Hospital Essen, Hufelandstraße 55, 45147 Essen, Christian.weimar@uk-essen.de, Representative of the DGN.

Jochen Wöhrle, Department of Internal Medicine II, University Hospital Ulm, Albert-Einstein-Allee 23, 89073 Ulm, jochen.woehrle@uniklinik-ulm.de, Representative of the DGK.

**For the Swiss expert association:**

Heinrich Mattle, Senior Consultant, Univ. Dept. of Neurology, Inselspital, CH-3010 Bern, Meisenweg 18, CH-3014 Bern, heinrich.mattle@bluewin.ch, Representative of the Swiss Neurological Society.

## Guideline creation methodology

The boards of the three participating scientific societies nominated authors to prepare the recommendations. Each of the five key questions was assigned to a team of authors comprising neurologists and cardiologists to create the text. The authors initially conducted a systematic literature search in Medline with the terms “stroke”, “cryptogenic stroke”, “patent foramen ovale”, “closure” and “randomised trial”. The search identified 32 hits in which 7 randomised studies were identified (Tables 1 and 2). This search was aligned with the published meta-analyses and guidelines (Table 3). The risk of bias was investigated in 3 meta-analyses [1, 2, 20] and due to the fact that the studies were not blinded (which was technically not possible) this was classified as minimal or not present. Endpoint adjudication was blinded in all studies. The meta-analysis of Mir et al. included the evaluation of studies according to GRADE criteria [21]. The referenced meta-analyses were published with one exception in peer-reviewed journals, which included methodologists competent in the review process. This was not the case in the meta-analysis of Wang et al. [22]. Therefore, this meta-analysis was not taken into account. The authors followed this assessment. The author groups then created the text sections and wrote the recommendations. The draft text was then proofread and corrected by three lead authors. Thereafter, the recommendations were read and corrected in three iterations from all members of the author group. Finally, the total text was signed off by a written Delphi procedure.

## Validity and update procedures

Date of last content revision and status: 13 June 2018.

Valid until: 12 June 2021.
