# Supplementary material for: Cryptogenic stroke and patent foramen ovale (abridged and translated version)
Source: Neurol Res Pract. 2019 Feb 28;1:1. doi: 10.1186/s42466-019-0008-2 (PMC7650129; doi:10.1186/s42466-019-0008-2)
Supplement: Supplementary file 1 — Statement of competing interests: tabular summary. (DOCX 22 kb) [file 42466_2019_8_MOESM1_ESM.docx]

## Additional file 1: Statement of interests: tabular summary

Originals of fully completed declarations of interest have been returned to the Guideline Coordinator/Editorial Office Guidelines (EO). For transparency reasons, all potential interests, even if not related thematically to the guideline, are presented in this table. If there is a link to the guideline, this will be mentioned. The final outcome of the assessments by a conflict of interest officer of the DGN and the guideline group is listed.

|  | Activity as Consultant or Expert Advisor | Contribution to a scientific advisory board (advisory board) | Lecturing and training activities | Authorship/Co-authorship | Planned research/Conduct of clinical trials | Owner interests in healthcare | Indirect interests | Thematic link to the guideline  Assessment of conflicts of interest and any associated corrective action |
| --- | --- | --- | --- | --- | --- | --- | --- | --- |
| **Hans-Christoph Diener (Coordinator, DGN)** | Abbott, Allergan, Bayer Vital, Bristol-Meyers-Squibb, Boehringer Ingelheim, BrainsGate, Daiichi-Sankyo, Medtronic, MSD, Pfizer, Portola, Sanofi-Aventis, Servier, St Jude | Abbott, Allergan, Bayer Vital, Bristol-Meyers-Squibb, Boehringer Ingelheim, BrainsGate, Daiichi-Sankyo, Lilly, Medtronic, MSD, Novartis, Pfizer, Portola, SanofiAventis, Servier, St Jude | Abbott, AstraZeneca, Bayer Vital, Bristol- Meyers-Squibb, Boehringer Ingelheim, BrainsGate, Daiichi- Sankyo, Lilly, Medtronic, MSD, Novartis, Pfizer, Portola, Sanofi- Aventis, Servier, St Jude | Publisher (*Aktuelle Neurologie* [Current Neurology], Info Neurologie und Psychiatrie [Info Neurology and Psychiatry], Lancet Neurology) | No studies on PFO closure | No | Membership in AAN, ANIM, Deutsche EEG- Gesellschaft [German EEG Society], DGN, DGSS, DSG, DEGUM, EHF, ESC, Headache Research Group, and many more Scientific activities: Stroke, headache: DGN Conference Employers: Essen University Hospital | Consultancy activity: no thematically relevant link of the information to the AdBoards guideline: For Abott [*sic:* Abbott], Medtronic, St Jude thematic link to the guideline (PFO and LAA closure systems), but without any financial contributions from industry lectures: for Abott [*sic: Abbott*], Medtronic, St Jude thematic link to the guideline (PFO and LAA closure systems), but apart from travel expenses (Abbott) without any further financial contributions from industry  No consequence |
| **Armin Grau**  **(Coordinator, DSG)** | No | No | No | Thieme Verlag (Textbook on metabolic disorders in neurology) | No | No | Memberships in DSG, DGN, AHA, AAN, DGKN Scientific activities: Stroke Clinical activities: General Neurology, mainly stroke  Employer: Hospital Ludwigshafen | No thematically relevant link of the information to the guideline  No consequence |
| **Stephan Baldus (Coordinator, DGK)** | Colemann Research Group,  Deutschlandradio [Germany radio], Doctrina Med, Jena Valve, Objective Focus, Aqua Institute (interventional cardiology in each case) |  | Abbott, Asklepios Hospital, Agapleson Allgemeine KH Hagen [Agapleson General Hospital Hagen], Astrazeneca Gmbh, Bayer Vital, Berlin-Chemistry, Bristol-Myers Squibb Gmbh, Boston Scientific, C.T.I. Conferences, Cardicon, Cardiovascular & Metabolism, Cme4u Gmbh, and many more (interventional cardiology in each case) |  | Abbott (IT MATTERHORN Study), Biotronik (Biovalve Heart Valves Study), Ico Vifor (EFFECT-HF Study), Jena Valve (AR EU Study, TAVI Study), Sanofi-Aventis Deutschland Gmbh (Registry Study), Optum Insight (Registry Study), Symetis (ACURATE neo study), Valtech | No | Membership of DGK, ESC Scientific activities: interventional valve therapy Clinical activities: Internal Medicine/Cardiology, Intensive Care/Interventional Cardiology Lead role: ESC Education Commitee [*sic:* Committee] Committee Employer: Cologne University Hospital | No thematically relevant link of the information to the guideline  No consequence |
| **Alexander Ghanem (Author)** |  |  |  |  |  |  | Employer: Asklepios Hospitals Hamburg/St Georg | No Interests  No Consequence |
| **Klaus Gröschel (author)** | Boehringer Ingelheim (Pradaxa) | Daiichi-Sankyo (Lixiana), Boehringer Ingelheim (Pradaxa), Medtronic (Reveal), Bristol-Meyers Squibb (Apixaban) | Boehringer Ingelheim (Idarucizumab), Bayer AG (Xarelto) |  |  |  | Employer: University Hospital Mainz | No thematically relevant link of the information to the guideline, active ingredients are not discussed in the guideline.  No consequence |
| **Christoph Liebtrau (author)** |  | Astra Zeneca (ticagrelor), ThermoFisher (biomarkers) | Astra Zeneca (ticagrelor), Bayer (rivaroxaban, Boehringer Ingelheim (dabigatran), Pfizer (apixaban), Neovasc (therapy-refractory angina), Elixir (bioresorbable scaffold) | Thieme (DUALE Reihe - Internal Medicine Textbook) |  |  | Membership in DGK (Interventional cardiology working group) Scientific activities: Interventional cardiology, biomarkers  research Clinical activities: Interventional Cardiology Lead role: Intervention cycle with the DGK  Employers: Kerckhoff-Klinik [Kerckhoff Hospital] Bad Nauheim, University Hospital Gießen/Marburg | No thematically relevant link of the information to the guideline  No consequence |
| **Steffen Massberg (Author)** | DGK (speaker of the clinical committee) | No | No | No | Roche (Multiplate analyser, co-funded a randomised study) | No | DGK Scientific activities: platelets, atherosclerosis, myocardial infarction Clinical activities: Interventional Cardiology Lead role: Director, Medical Department and Outpatient Department I, University Hospital Munich: Ludwig-Maximilian University Hospital Munich | No thematically relevant link of the information to the guideline  No consequence |
| **Heinrich Mattle (Author, CH)** | Swissmedic (HPV vaccination and multiple sclerosis) | Bayer, Healthcare, Biogen, Boston Scientific, Daiichi Sankyo, Medtronic, Neuravi, Novartis, Pfizer, Sanofi, Servier (advisor for each), Research Council of Norway, and several research foundations in France and Spain (expert) | None with compensation | Cf PubMed under Mattle H | Neuravi, Medtronic, MR CLEAN LATE, Servier | No | Memberships in SHG, ESO, WSO, ASA, SNS, AAN Scientific Operations: Cerebrovascular disorders Clinical activities: General neurology, cerebrovascular disease, multiple sclerosis Lead role: text books, guidelines Employer: Inselspital Bern | No thematically relevant link of the information to the guideline  No consequence |
| **Helge Möllmann (author)** |  |  | SJM (heart valves), Abbott (heart valves, stents) | None on the topic of POO closure | None on the topic of POO closure | No | Membership of DGK Scientific activities: Interventional Cardiology Employers: St Johannes Hospital Dortmund | No thematically relevant link of the information to the guideline  No consequence |
| **Holger Nef (Author)** | Abbott (ABSORB, OCT) | Boston Scientific (Promus Stent, Acurate Nei), STENTYS (Stentys stent) | Medtronic (Corevalve, Evolute), Abbott (OCT, ABSORB) | None on the topic of POO closure | ATLANTIC, APPOSITION III; GREAT; OPEN II; RESTORE II; DEUS; RE-ADAPT-HF; Global Leaders, DESolve PMCF, and many more |  | Employer: University Hospital Gießen/Marburg, Cardiovascular Centre Rotenburg a.d. Fulda | No thematically relevant link of the information to the guideline  No consequence |
| **Dirk Sander (author)** | Bavarian State Chambers of Physicians (expert opinion on the topic of MS) | No | Pfizer (secondary stroke prevention); company without relevant products for guideline) | None on the topic of POO closure | None on the topic of POO closure | No | Scientific activities: stroke, atherosclerosis Employer: Benedictus Hospital Tutzing | No thematically relevant link of the information to the guideline  No consequence |
| **Christian Weimar (author)** | No | Bayer (rivaroxaban), BMS (apixaban) | Daiichi (edoxaban), Penumbra (thrombectomy catheter), Biogen (dementia), Boehringer (PRODAST Registry), AMGEN (evolocumab), OmniAmed (DOAC) | No | Boehringer (dabigatran), Johnson & Johnson (dementia research) | Bayer, Merck, MSD | Membership of DGN Scientific activities: Stroke, cognition Clinical activities: Vascular Neurology  Employers: Essen University Hospital | No direct, thematically relevant association of information with the guideline, but due to interests in the cerebrovascular sector abstention on DOACs (SF 2/Recommendation 2) |
| **Jochen Wöhrle (author)** | No | No | No | No | Bayer Healthcare (Galileo Study - TAVI), Boston Scientific (Lotus - TAVI) | No | Membership of DGK Scientific activities: TAVI heart valves, chronic coronary occlusion Clinical activities: Therapy of coronary or structural heart diseases Lead role: Professional development at the University Hospital Ulm Employer: Ulm University Hospital | No thematically relevant link of the information to the guideline  No consequence |
| Overall assessment of the guideline group in relation to the 50% rule of the DGN:  There was compliance with the 50% rule of the DGN (i.e. at least half of contributors must have no or only minor conflicts of interest in relation to the topic, with relevance for the guideline). | | | | | | | | |
